# The Effects of Stigma on the Caregivers of Elderly Patients With Psychiatric Issues in a Chinese Community

**DOI:** 10.1093/geroni/igab046.979

**Published:** 2021-12-17

**Authors:** Joanne Siu Ping So, Kai Nin Joseph Chan

**Affiliations:** 1 University of Victoria, Canada; 2 Asia Pacific Career Development Association, 20850, Maryland, United States

## Abstract

World Health Organization in 2017 indicates the proportion of persons over the age of 60 years will exponentially grow from 12% in 2015 to 22% in 2050. Advanced age is a common risk factor for multiple conditions, including psychosis, depression, and other mental illnesses linked to cognitive and neurologic disorders. The majority of the studies identify ethnicity and socioeconomic status as primary determinants of mental health care access. Recent studies show that up to 12% of elderly Chinese have had a history of mental problems. However, over 50% of Chinese with mental disorders have failed to obtain professional help. Lack of access to health care for mental disorders has been linked to multiple underlying socioeconomic and cultural factors. These Chinese Americans lack an in-depth understanding of their psychosis, and psychiatric conditions are often a minority in nature. This study will systematically review the existing situations relating the factors to the stigma on caregivers. The result shows that the leading cause of psychiatric disorders, physical and emotional components of the elderly population, needs to be incorporated in the care plan in nursing homes and hospitals. In North America, the constant perception of discrimination and the inherent feeling of isolation and stigma among families with elderly members remains challenging. This review could contribute to the policy reform, which can help design effective control strategies to manage gaps of most mental disorders that continue to disproportionately affect different ethnic groups across the U. S. and Canada.

